# Adult Daughters of Alcoholic Parents—A Qualitative Study of These Women’s Pregnancy Experiences and the Potential Implications for Antenatal Care Provision

**DOI:** 10.3390/ijerph19063714

**Published:** 2022-03-21

**Authors:** Helle Johnsen, Mette Juhl, Bodil Kirstine Møller, Vibeke de Lichtenberg

**Affiliations:** Department of Midwifery and Therapeutic Sciences, University College Copenhagen, Sigurdsgade 26, 2200 Copenhagen N, Denmark; meju@kp.dk (M.J.); bodilkirstine1@gmail.com (B.K.M.); vide@kp.dk (V.d.L.)

**Keywords:** children of alcoholics, pregnancy, antenatal care

## Abstract

The adult children of alcoholic parents are at increased risk of having health problems compared to the adult children of nonalcoholic parents. Little is known about how growing up with alcoholic parents affects women’s experiences when pregnant. The objectives of this study were to explore how adverse childhood experiences related to parental alcohol abuse affect women during their pregnancy and to assess the potential implications of women’s experiences for antenatal care provision. Twelve in-depth interviews were performed with women who were brought up by an alcoholic mother and/or father. Systematic text condensation was used to analyse the data. Two main categories were identified: ‘establishing relationships and having social support’ and ‘antenatal care encounters and concerns during pregnancy’. Women’s trust in others in adult life was impacted by their upbringing. Strained relationships with their parents and few friends meant that the women primarily relied on their partners for support. Neither antenatal care providers nor women talked about women’s childhood experiences at the visits. The women described concerns related to the baby’s health, lack of predictability and control during the pregnancy, as well as apprehensiveness regarding birth and motherhood. The potential implications for practice include systematic screening for adverse childhood experiences, antenatal preparation classes, parenting courses, and post-graduate training.

## 1. Introduction

In Denmark, it is estimated that 122,000 children (10.6% of the population) below eighteen years of age grow up in families with alcohol problems [1]. Being the child of an alcoholic parent is associated with a lower educational level and lower economic capability in adulthood [2]. Compared to young people (ages 12–25) without perceived parental alcohol problems, young people with perceived parental alcohol problems have been found to have higher odds of internalising problems, frequent emotional symptoms, and poor parent relationships, such as a lack of parental interest [3]. Young people (ages 12–25) with perceived parental alcohol problems have also been found to have higher odds of self-injury, suicidal ideation, and suicide attempts, compared to young people without perceived parental alcohol problems [4].

Traumatic childhood experiences may also affect individual health in adult life. Stu-dies have shown that adults who have grown up with an alcoholic parent are at increased risk of mental illness, poorer perceived health, and cardiovascular disease, compared to adults having grown up with nonalcoholic parents [5,6,7]. Furthermore, studies over the last two decades have consistently shown that growing up with an alcoholic parent is likely to be accompanied by other adverse childhood experiences, such as psychological and physical abuse, domestic violence, and neglect [6,8]. Sustained trauma commencing in early childhood can change neurobiological integration and processing, which affects the mechanisms that help to cope with stressful stimuli and regulate emotion; it can also predispose individuals to vulnerability to further harm, as well as inter- and intra-personal difficulties [9]. Types of trauma can be categorised by the nature of traumatic events, the experiences of these events, and/or the effects of these events [9]. In the case of parental alcohol abuse, studies have pointed to the risk of transmission of alcohol abuse between generations. A study investigating possible links between maternal alcohol disorder abuse and early alcohol use in offspring has shown that maternal alcohol use disorder can lead to financial strain and depressive symptoms, which can produce a negative family climate [10]. This climate may subsequently increase the likelihood of alcohol use in offspring by the age of 15. Another study has shown that alcohol use during adolescence is a predictor of family formations in which both the mother and father abuse alcohol, which in turn increases the likelihood of offspring experimenting with alcohol during childhood and the use of alcohol by early adolescence [11]. These studies highlight the importance of identifying individuals with childhood experiences of alcoholic parents as early as possible in life.

Approximately one out of five families in Denmark experiences problems related to a lack of parental resources and neglect [12]. Research has shown that compared to the adult sons of alcoholic parents, adult daughters are at increased risk of experiencing mental health problems and poor health generally [5]. For women who have grown up in a dysfunctional home, pregnancy and early motherhood pose a particularly challenging period [13,14]. Having two or more adverse childhood experiences (ACEs) encompassing emotional, physical, and sexual abuse, as well as family dysfunction, such as exposure to domestic violence and parental substance abuse, are associated with higher odds of having an unwanted pregnancy [15]. In addition, a positive association between ACEs and antepartum health risks (prepregnancy risk factors, past obstetrical risk factors, problems in the current pregnancy, and other risk factors) has been documented [16]. ACEs are also associated with the risk of depression during pregnancy as well as after birth [17,18]. After birth, experiences of childhood relational trauma in a parent may be replicated in their children through the attachment relationship, because of the relational nature of early childhood neurobiological development [9]. Furthermore, neglect during childhood has been shown to lead to negative parenting behaviour [19], which can increase the risk of insecure or disorganised attachment patterns in offspring [12].

At the same time, pregnancy is considered a transformative period [20] and, thus, offers an important window of opportunity for intervention among mothers-to-be [21]. Currently the research on the adult children of alcoholics is limited and mainly focuses on the health risks associated with growing up with alcoholic parents in adults. Less is known about the daughters of alcoholic parents during the pregnancy period. Furthermore, research investigating the significance of these women’s pregnancy experiences for the provision of targeted antenatal care is currently lacking. This scarcity, especially in terms of qualitative studies, calls for further research.

The objective of this study was to explore how adverse childhood experiences related to parental alcohol abuse affect women during their pregnancy and to assess the potential implications of women’s experiences for antenatal care provision. The paper starts by exploring women’s descriptions of their upbringing, their adult life, and current pregnancy experiences. The subsequent discussion examines the significance of these findings for the provision of targeted antenatal care (ANC).

### Antenatal Care in Denmark

Danish ANC is publicly funded and free of charge [22]. The Danish National Health Act stipulates that women are entitled to receive at least five free ANC visits [23]. Danish ANC is differentiated and divided into four levels, ranging from basic ANC provision to extended ANC provision involving interdisciplinary cooperation [22]. Women’s physical and psychosocial health is initially assessed by the general practitioner at the first ANC visit, at which point the general practitioner also considers whether specialised care is required. In uncomplicated pregnancies, the midwife is the maternity care provider the woman sees the most.

In Denmark, there is no systematic monitoring of the utilization of ANC by different groups of women. The utilisation of ANC services partially depends on how ANC is organized in the different Danish regions, as well as at local maternity wards. A recent study has documented that the number of midwifery visits offered to women and the duration of these visits vary greatly between Denmark’s 20 maternity care wards [24].

## 2. Materials and Methods

A qualitative study design [25] was chosen to illuminate women’s experiences of being daughters of alcoholic parents during their childhood and adulthood, including the pregnancy period.

### 2.1. Recruitment and Collection of Data

Purposeful sampling was used to recruit women from a non-governmental organisation (NGO), which provided counselling and therapy for teenagers and adults who had grown up with alcoholic parents. Inclusion criteria were growing up with one or two parents with alcohol abuse and not currently being diagnosed with depression or any other serious mental illness.

In-depth interviews were performed to allow sufficient time to explore the women’s experiences [25]. Interviews were performed face-to-face. A semi-structured interview guide [26] was used to collect data. Interviews were performed by Vibeke de Lichtenberg (VL). The main questions in the interview guide were centred around women’s relationships and experiences with their parents during childhood and in adult life, relationships and experiences with significant others, how women reacted to expecting a child, and their experiences of their encounters with ANC providers. The interviews had a duration between one hour and forty minutes and two hours and twelve minutes. The average interview duration was one hour and fifty-five minutes. Women selected the date, time, and location for the interview. All interviews were audio-recorded and were subsequently transcribed verbatim.

### 2.2. Data Analysis

**Figure 1:**
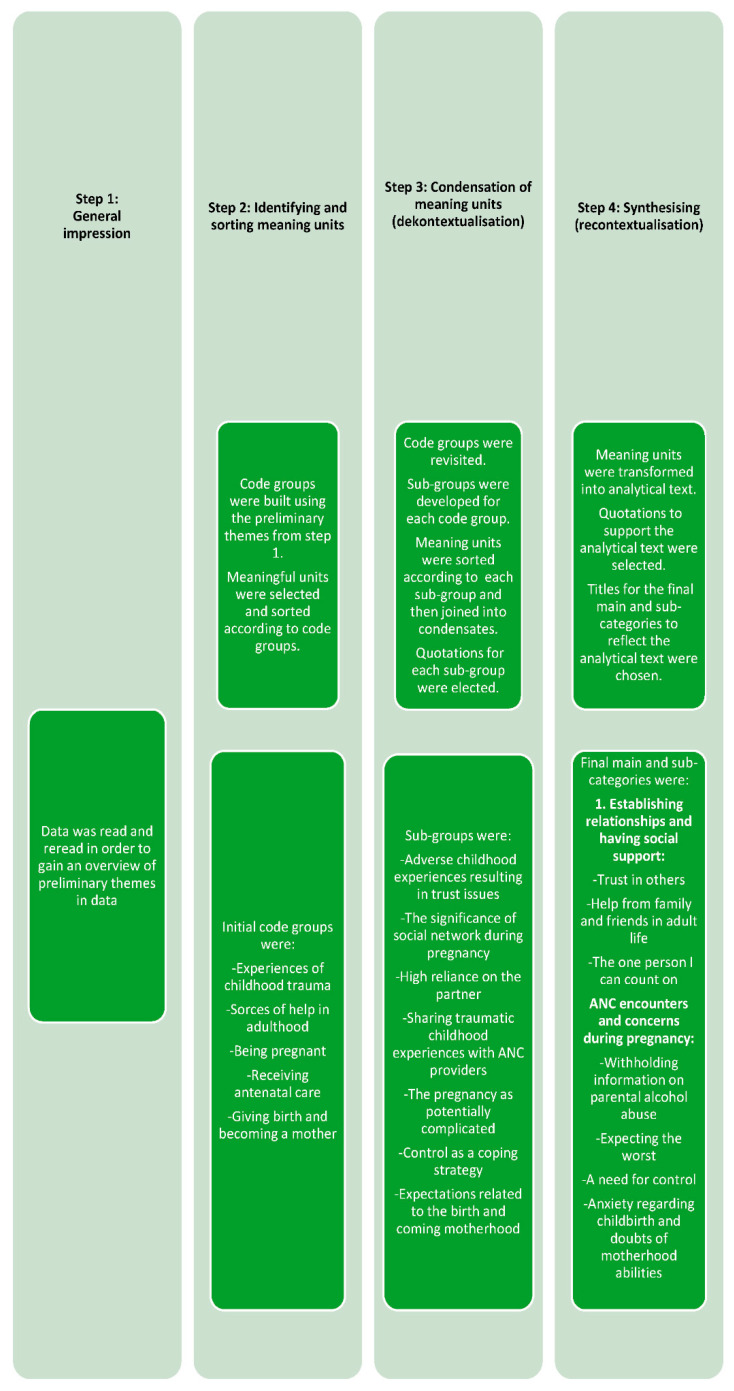
The analysis process.

Data were analysed using systematic text condensation, as described by Malterud [27]. This method consists of four steps. In Figure 1, the different analytical steps are explained in more detail. Although it is described as a linear process in the figure, the actual analysis process was more dialectical due to the continuous movement between the differ-rent steps in the analysis. This meant that as the analysis of the data evolved, the original data were revisited to refine and modify existing categories. Helle Johnsen (H.J.) undertook analytical steps one and two. Authors H.J. and V.d.L. undertook analytical step three. All authors participated in step four of the analysis, which included discussing the analysis to ensure that the final categories and sub-categories were grounded in women’s descriptions and covered the entire dataset.

### 2.3. Ethical Considerations

Women received written and verbal information about the study before verbally consenting to participate. They were guaranteed personal anonymity. To ensure women’s anonymity, all names were removed from the results section, and women are identified according to the number of the interview. Prior to giving consent to participate in the study, women were informed that they could withdraw at any time, should they wish to do so.

Growing up with alcoholic parents is a sensitive issue, especially when expecting a child of your own. The interviews were carried out by V.d.L., who is a certified psychotherapist and has numerous years of professional experience working with women with different psychosocial vulnerabilities. Furthermore, all women participating in the study were recruited from an NGO, which provided counseling and therapy for adults who had grown up with alcoholic parents. Thus, participants had access to help in case they found the interview topics distressing.

In Denmark, certain types of research projects must be reported to and approved by a research ethics committee. This applies to clinical trials and studies that involve human biological material. According to the National Committee on Health Research Ethics, studies using interviews that do not involve human biological material (i.e., the present study) should not be reported to this committee (section 14(2) of the Committee Act) [28].

The study, including a description of measures regarding data protection, was reported to:

The Research, Development, and Data Department, University College Copenhagen (ID number: 21-009).

## 3. Results

In all, 12 women were included in the study. Seven of the women grew up with an alcoholic father. Five of the women grew up with both an alcoholic father and an alcoholic mother. Two women had parents with other types of substance abuse in addition to alcoholism. The women’s ages ranged from 25 to 38 years. Ten women were expecting their first child and two their second or third child. The women were between 34 and 39 weeks pregnant. The highest educational levels among the women were college (*n* = 1) and graduate university level (*n* = 11). Eleven women were cohabiting with a male partner.

The analysis of data revealed two main categories with three and four subcategories, respectively. The main categories were ‘establishing relationships and having social support’ and ‘ANC encounters and concerns during pregnancy’.

### 3.1. Establishing Relationships and Having Social Support

This category describes how having alcoholic parents affected women’s confidence in others, as well as the character of women’s social relations during their childhood and in adult life.

#### 3.1.1. Trust in Others

Growing up with alcoholic parents was described as a lonely situation by most of the women. When a parent drank, it greatly affected the home environment. The women described how they would attempt to withdraw from situations in which their parents were intoxicated, such as by hiding in the garden (I12) or walking the dog (I7). Some women had tried to confront their parents about their abuse; however, their parents had not listened to them and had not considered their behaviour problematic to the same extent as the women did. Some parents also stated that they felt that as long as their children had food, clothing and a place to live, they had received adequate care. A few of the women described serious neglect during their childhood, such as being left alone at home, not having anything to eat and not being taken to the doctor when sick.

In general, the women described their childhood circumstances as unpredictable and their parents as unreliable. Daily life was governed, to a large extent, by the severity and character of the parents’ drinking habits. Most of the women had learned to take responsibility and to take care of themselves and, sometimes, siblings, at a very early age. These conditions led to mistrust towards their parents. Inexperience with stable, trusting relationships with their parents also led to mistrust towards others.. Several of the women described having few or no friends. One woman described, how she, as a child, had tried to avoid rejection by others by not reaching out to others [I4]. Although a lack of friends was described as problematic by the women, it also served to protect them from potential risks of becoming disappointed or being abandoned. This was a strategy that the women continued to make use of in their adult life:

“It has affected me a lot. I have always kept people at a distance … even friends I have had since my childhood … I have never allowed myself to feel 100% sure about anyone …”

(Pregnant woman, interview 1)

Several women described themselves as having low self-esteem and self-worth, both as children and in adult life. These feelings resulted in their never feeling good enough. These women described themselves as introverted and as having difficulties expressing how they felt. Furthermore, some women continuously reflected on what other people thought of them:

“… I can see how my behaviour is affected by my upbringing… being afraid of being rejected by my husband, anyone, my friends … they probably don’t want to be with me … I won’t contact them … It’s insecurity in myself … am I good enough? … will people like me?”

(Pregnant woman, interview 11)

#### 3.1.2. Help from Family and Friends in Adult Life

Several of the women described how they lacked a social network in adult life. A few of the women had lost a parent due to their alcohol abuse. Others explained that they had cut family ties with their parents because of their childhood experiences. Among the women who had maintained relationships with their parents, many relationships were depicted as strenuous because their parents had continued their alcohol abuse and lived unstable lives. These women described themselves as being parents for their parents. Furthermore, some women reported that they maintained contact with their parents for their parents’ sake rather than their own. Several of the women had grown up with siblings. However, like the women themselves, their siblings had grown up under turbulent and insecure circumstances. A few of the women described how a sister or a brother had also become an alcoholic and how they would try to support them. As a result, several of the women lacked emotional and practical support from their immediate family in adult life. These circumstances intertwined with the women’s pregnancy experiences and their expectations following birth. The women were unsure whether they could rely on their families, especially their mothers. Living an adult life perceived to be very different from their mothers also meant that the women, to a lesser extent, drew upon their mothers for informational and emotional support during their pregnancies.

Having other social relations was also described as difficult. Some of the women explained how their friends had grown up in more well-functioning homes and, consequently, they perceived their friends’ competencies as very different from their own. In general, the women described their childhood experiences as greatly affecting their social competencies:

“… I have never been very social. I have always been somewhat socially handicapped … if you feel something is wrong with you at home, you feel the same in other relationships. I have never been very good at having many relationships and friends … I have very few friends and prefer someone I can trust …”

(Pregnant woman, interview 8)

Among the women who described having close female friends, there seemed to be a reluctance to use these friends for support. Furthermore, because these women had numerous experiences of broken promises as children, some described themselves as constantly monitoring whether their friendships were worth continuing in adult life:

“I am probably not as well balanced as other people … I am very black-and-white, so to speak, if you have plans together, you stick to them … If people don’t follow through on their plans with me … I won’t put up with it … I quickly cut people off if they don’t keep their promises …”

(Pregnant woman, interview 6)

#### 3.1.3. The One Person I Can Count On

The women found their partners to be the most important source of support. The partner was described as an important source of practical help at home. He stepped in when the pregnancy prevented the women from participating in domestic tasks. The partner also helped with preparing the home for the baby by, for example, preparing the nursery. Because the women drew to a lesser extent upon their parents, siblings, or friends for emotional support, their partner was described as key for their mental well-being during the pregnancy. In general, the women explained that their partners knew about their upbringing. Several women highlighted that their partner came from a home with more sound family relations and, thus, was able to offer them the stability, reliability and emotional support they had missed growing up:

“… the most important person is without a doubt my boyfriend! He has put up with a lot … (he) is definitely the person who knows me the best and the best support I have now.”

(Pregnant woman, interview 9)

The women also drew upon their partners when they became nervous, or were uncertain about how to react to symptoms during their pregnancy. Some women described how it was difficult for them to navigate between overreacting and reacting appropriately to unexpected pregnancy circumstances. In these situations, the partner would help calm them and help them to approach the situation more rationally:

“… I talk to my husband a lot (about the pregnancy) … He has the capability to encompass all situations (concerning the pregnancy) … I can see they (my pregnancy concerns) tend to be silly. I am so blessed I have him. He is very down-to-earth and doesn’t worry so much.”

(Pregnant woman, interview 8)

“… he tells me: ’It’s good you tell me, I want you to tell me if you are worried’. So, I have used him a lot. He tries to calm me.”

(Pregnant woman, interview 10)

### 3.2. ANC Encounters and Concerns during Pregnancy

This category illustrates how the women’s traumatic childhood experiences were addressed by doctors, midwives and the women themselves during the ANC visits. The category also describes how the women’s childhood experiences affected their pregnancy expectations, their attempts to control their pregnancy and their apprehensions regarding the birth and approaching motherhood.

#### 3.2.1. Withholding Information on Parental Alcohol Abuse

As children, most of the women had tried to keep their parents’ alcohol abuse a private matter. This meant that they withheld this information from their friends, teachers and other formal contacts. Hiding parents’ alcohol abuse continued into adult life, where having alcoholic parents was associated with stigma and potential prejudice from others:

“… It’s kind of stereotype … connected to the stigma, being the child of an alcoholic … what other people think about this. I have been very private about it. A lot of people don’t know. I need to be very close to a person before I have the courage to tell them … Some are very open about it … I think it is very embarrassing, even though it is not my fault … I don’t want people to judge me because of it.”

(Pregnant woman, interview 5)

In general, discussing their parents’ alcohol abuse with health care professionals during childhood and in adult life was described as very rare. This was also the case during the ANC visits. If the women were not directly asked about their upbringing at their visits, they would not volunteer this information. One woman explained how she was surprised that she had not been asked about her childhood during her visits:

“I talked to my boyfriend about it. Odd they didn’t ask me about this … In fact, they haven’t talked about it (my upbringing) at any point during my ANC visits.”

(Pregnant woman, interview 10)

Out of the 12 women, one woman had discussed her childhood vulnerabilities with her ANC provider.

“… it is written in my antenatal record that I am psychologically vulnerable… My doctor wrote it in case anything turns up … it’s nice that it is recorded in my record.”

(Pregnant woman, interview 8)

The women described their childhood experiences as having affected how they felt during the pregnancy. However, the women were not prone to discuss these feelings with ANC providers. The womens’ conversations with ANC providers mainly focused on their physical situation. The women described talking to their ANC providers about subjects such as adequate food intake, weight gain and their baby’s growth.

Some of the women also highlighted that changing carers, preset agendas for the ANC visits, and short appointments, affected their motivation to discuss how they felt:

“… I haven’t talked to my midwife (about how I feel) … I have only seen her twice … The last visit regarded breastfeeding … this did not reflect my needs … breastfeeding is a long time away … There is not a lot of time during the antenatal visits. It is very much like in and out (of the door).”

(Pregnant woman, interview 6)

#### 3.2.2. Expecting the Worst

Although the women perceived concerns over their baby’s health to be a common phenomenon among pregnant women, some women also described an alertness that went beyond general pregnancy-related worries. A few of the women reported that they had been very nervous about whether something was wrong with the baby (I5, I8). Another woman described how she was afraid about whether her food intake was sufficient for the baby to grow:

“I am very aware of my food situation… that I get enough to eat and that she (the baby) gets enough … It’s something I can feel preoccupies me a lot.”

(Pregnant woman, interview 2)

For the women, growing up with alcoholic parents had been one of several traumatic childhood experiences. Circumstances such as parental illness and, in a few instances, death, domestic violence and unstable housing conditions meant that some of the women were prone to expecting poor outcomes in adult life. Thus, expecting a child meant that some women also expected the worst during pregnancy:

“… I am by nature or as a result of everything (my childhood experiences) very much a catastrophe thinker. I think of all the things that can go wrong … a month ago … I had a tendency to have high blood pressure … it was very uncomfortable because, as expected, something was wrong and my catastrophe thoughts started rolling.”

(Pregnant woman, interview 9)

“… I think … I bring bad fortune. If something can go wrong, it probably will … first I had to get through the ultrasound examinations … then all the worries began.”

(Pregnant woman, Interview 11)

“I have catastrophe thoughts … It is something I have to battle … Everything will go wrong … it’s not very nice (to feel like that).”

(Pregnant woman, interview 12)

#### 3.2.3. A Need for Control

The need for a high level of control characterised several of the women’s adult lives. The women described how, during childhood, they had tried to cope with instability by controlling the elements they were able to control. Being in control gave the women predictability. This also meant that elements that were uncontrollable in adult life were perceived to be very challenging:

“I have an enormous need for control … because of my father’s problem (his drinking) … I need things done my way … I can be really hard on myself, which also originates from my childhood … I am also the person who constantly takes responsibility and thinks ahead.”

(Pregnant woman, interview 12)

“I prefer a high level of control … so when I lose control, I don’t always handle it well … as a child I was always in control of what happened at home because I, to a large extent, took care of things.”

(Pregnant woman, interview 11)

During the pregnancy, a high need for control was also described by the women. Having control served as a strategy for the women to cope with being pregnant. Several of the women described exerting a high degree of self-discipline during the pregnancy period. For example, they abstained from alcohol, went to great lengths to assure a healthy diet and continued to exercise throughout their pregnancy. Some of the women found it problematic when the pregnancy prevented them from adhering to normal routines, such as continuing their work–life or domestic responsibilities. Furthermore, being pregnant was considered to be quite challenging by some of the women. This was due to the fact that the pregnancy was perceived to be less controllable, which created uncertainty:

“It’s quite anxiety-provoking that I am in the middle of something (being pregnant) that I am not able to control in any way. I don’t know what is ahead and that is really difficult.”

(Pregnant woman, interview 8)

Unexpected situations were described as especially difficult to manage. Having time to prepare for a situation was perceived as necessary:

“… I need to prepare myself mentally for how to handle things. I have a really hard time when things change and I am unprepared…”

(Pregnant woman, interview 5)

During pregnancy, conditions were different and, while some aspects related to the pregnancy were controllable, others were not. One woman described how she felt she had lost control over her body:

“It has been a great challenge for me to accept that there is something in my body I can’t control, something that grows, and that has its own life…”

(Pregnant woman, interview 7)

Some of the women found it difficult when they were not able to perform the same way as before they became pregnant, such as meeting usual job demands and sticking to their exercise programs. Furthermore, not being able to control bodily changes during pregnancy was perceived as difficult. These difficulties were especially related to gaining weight. One woman described how weight control had been a central part of being a teenager and, later, an adult:

“… my bodily change, the physical change…I have to weigh myself during pregnancy…my weight has been something I have previously been able to control…It triggers my need for control … I need to put on weight, this makes it really difficult…”

(Pregnant woman, interview 8)

#### 3.2.4. Anxiety Regarding Childbirth and Doubts of Motherhood Abilities

While the women described numerous reflections related to being pregnant, having a plan for the birth was less pronounced among the women. Some of the women participated in antenatal preparation classes. These classes were described as beneficial as they increased knowledge about pregnancy and birth and equipped the women to make informed choices. The women enjoyed meeting other pregnant women and sharing experiences. Furthermore, some of the women described how antenatal preparation classes had provided them with different tools to manage birth.

“… you meet other first time mothers and you are able to share and talk about your concerns…you also get different tools to help you breathe and relax … it has given me more confidence that I have acquired knowledge and experience…”

(Pregnant woman, interview 9)

At the same time, several women reported being anxious about their ability to cope with birth and concerns about their baby being hurt during the birthing process. Several of the women described sharing their birth expectations with their partners. In some instances, they also discussed their expectations with a family member or a friend. However, only a few women discussed their birth expectations with their ANC providers. This meant that some women primarily tried to process their expectations of the upcoming birth on their own:

“… I used to look forward to the birth but now where it gets closer, I get somewhat nervous … I haven’t talked to anyone about it…”

(Pregnant woman, interview 1)

“I mostly look forward to when it’s over (the birth), but I also fear it … When you go into labour, you can’t stop it, and you don’t know what it is like before you try it … it’s extremely hard for me.”

(Pregnant woman, interview 8)

As birth could be related to negative expectations, some women tried to ignore it:

“I try not to think about it, because if I do … it gets out of control … so the less I think about it the better it will be … I haven’t talked to my midwife about this…”

(Pregnant woman, interview 4)

Instead of reflecting on the upcoming birth, several of the women described focusing their reflections on the time after birth. Some of the women saw the arrival of their child as a fresh start in life. The women looked forward to seeing and holding their baby and, together with their partner, being a family. Several women explained how it was very important for them to ensure that their child did not grow up in a malfunctioning family. At the same time, they also saw the time after birth as potentially difficult and uncertain. Some of the women described having doubts about whether they were emotionally strong enough to take care of a baby. One woman described how her childhood experiences affected her confidence in becoming a mother:

“I have been concerned about whether I will be a good mother. Am I carrying too much emotional baggage which will affect the baby?… It makes me upset because it is really hard … As a person, my mood varies a lot … Will I become a parent like my parents?”

(Pregnant woman, interview 3)

Another woman expressed concerns about the changes and potential risks a child would bring:

“I have been extremely mentally affected by the pregnancy … more than I had expected … even though this is a planned pregnancy … I am really worried about everything changing … that things are unpredictable … that I will see a shift in my identity … What if my boyfriend and I split up? What if we can’t handle it … Your whole existence changes, when you become a mother … I can accept that I am expecting a baby, but I have more difficulties imagining myself as a mother…”

(Pregnant woman, interview 12)

## 4. Discussion

The findings from this study showed that the women described themselves as having difficulties with establishing relationships and generally lacking trust in others. In addition, the women saw themselves as having low self-worth and self-esteem, which decreased their confidence in communicating problems and concerns to their ANC providers. Similar characteristics were documented in a study showing that the adult children of alcoholics are liable to have problems regarding self-confidence and feelings of insecurity [29]. This study also showed that an inability to cope with uncertainty contributed to personal stress in adult life.

Furthermore, besides having a partner, the women in this study tended to lack a social network. As a result, they were extremely dependent on their partners. The women’s partners were generally described as very important sources of social support. Neither the women nor their partners dealt with any forms of alcohol abuse. However, other studies have shown that alcohol abuse may be transferred between alcoholic parents and their children [10,11]. In addition, women with excessive intake of alcohol during adolescence and adulthood are more likely to have partners with similar histories of alcohol abuse [11]. The women in this study described having few female friends, which resulted in decreased informational and practical support from people other than their partner. This added to their social vulnerability. Being socially vulnerable has been shown to impact access to, as well as experiences of, ANC. A previous study has documented that socially disadvantaged women are less likely to use ANC services and more likely to have negative experiences when interacting with their midwives and doctors [30].

Interestingly, only a few of the women described their ANC providers as important sources of formal support during pregnancy, even though they lacked informal social support. Previous studies documented the importance of social capital and social support during the pregnancy period. A recent study points to the importance of social capital for women’s mental health during pregnancy [31]. Another study showed that antenatal health risks among women with a history of ACEs may be decreased if women experience high levels of social support during their pregnancy [16]. Furthermore, having social support during pregnancy is associated with decreased risk of depression during pregnancy and after birth [17,18].

Although several of the women in this study had contact with their parents, these relations were also described as strained. Due et al. argue that social relations may also feature negative dimensions, such as high demands from family relations and conflict [32]. Järvinen et al. argue that the adult children of alcoholics may have different approaches to understanding alcoholism [33]. When perceiving parents’ alcohol abuse as a disease, adult children learn from an early age to take care of their parents. When regarding parents’ alcohol abuse as a volitional behaviour, adult children tend to distance themselves from their parents through deliberate choices and willpower. In this study, rather than adhering to one approach, the women perceived their parents’ behaviour both as a disease and as volitional. Some of the women described themselves as becoming parents for their alcoholic parents. At the same time, they also exerted great self-control in their adult life, including during their pregnancy period.

In line with previous research [6,8], several of the women in this study described having experienced more than one type of adverse childhood trauma. The women also reported that their ANC providers did not ask them about their upbringing and the women themselves would not convey this information if they were not asked. This may have been because the women were used to hiding their parent’s alcohol problems to protect them. Moreover, the women may have been reluctant to report this information, due to their concerns over personal stigma.

Shim uses the term cultural health capital to refer to potential patient attributes, such as linguistic competencies, a proactive attitude toward accumulating knowledge, the abi-lity to understand and use biomedical information and showing interest in personal health and initiative in self-care [34]. She argues that these attributes are especially in demand in Western health care systems. It is likely that the women in this study had high levels of cultural capital in their encounters with ANC providers. For example, the women exerted high levels of self-discipline regarding diet, exercise and weight gain. Although these traits can promote foetal health, they may also, in very serious cases, be related to compulsive behaviour, which may require help from a psychologist or psychiatrist.

Several of the women in this study described high levels of concern for their baby. A previous longitudinal study showed that the main concern women have throughout their pregnancies is worries about foetal health [35]. While concerns for the baby are liable to be common among all pregnant women, a recent cross-sectional study by Eide et al. has showed that compared to women with no abuse during childhood, women who experience childhood abuse have an increased risk of strong worries about their baby’s health [36]. This study also found that women’s distrust of ANC was associated with significant worries about the baby’s health [36].

In addition, the findings showed that several women were unprepared for the upcoming birth. This was despite the fact that the women were close to their due date. Some of the women participated in antenatal preparation classes and found these helpful in increasing their knowledge about the upcoming birth. At the same time, several of the women were uncertain about the time after birth, including their ability to bond with their baby as well as the changes a child would bring to their relationship with their partner. This may have been due to a lack of healthy parental role models during their childhood.

The Danish antenatal care recommendations stipulate that, in order to determine whether women should receive basic or extended ANC services, a comprehensive record of women’s medical history should be collected [22]. Most of the women in this study were placed in level one of ANC, which entails basic ANC, including fewer visits than in higher levels of ANC. According to the women’s antenatal records, they were educated, had employment, and were cohabiting with a partner. These factors may have decreased the care providers’ attention to the women’s psychosocial vulnerability. A recent study showed that adverse childhood experiences among pregnant women are liable to be present in adult life regardless of women’s social class, economical position, or educational level [16]. Thus, vulnerability assessments based on women’s socio-economic status risk overlooking psychosocial vulnerability in more affluent populations.

The World Health Organization (WHO) highlights the importance of traumatic childhood experiences for lifelong individual health and well-being [37]. It also recommends systematic screening for these experiences in health care services through the use of the Adverse Childhood Experiences questionnaire (ACE) [8], which that can identify family dysfunction during childhood, including physical, sexual and emotional abuse and neglect by parents or primary caregivers. Previous studies have shown that a maternal history of adverse childhood events can be used as a predictor for risk of depression, anxiety, antepartum health problems and partner violence [15,16,17,38], suggesting the possible benefits of screening for childhood trauma among all pregnant women upon entrance to ANC.

Furthermore, there is a need for ANC providers to be aware of traumatic childhood experiences to understand women’s concerns during pregnancy, as well as their reactions to potential pregnancy complications. Without this knowledge, women’s concerns and reactions risk being labelled as normal or, potentially, exaggerated. A previous systematic review and meta-analysis showed that antenatal preparation classes may be effective at reducing fear of childbirth [39]. As the women in this study were concerned about their ability to cope with the birthing process, these classes are potentially helpful for this group of women. At the same time, this type of preparation may not offer enough support for these women after birth, since they may need preparation for the emotional challenges that may occur after birth. Several parental courses have been shown to be effective, including the ‘Circle of Security’, a training method aiming to improve how mothers respond to their children and cope with parenthood [40].

Organisational factors related to a limited number of antenatal visits, time restrictions, and antenatal care providers’ high task loads impacted the women’s ANC encounters negatively. The WHO recommendations for ANC stipulate that ANC should take place in a well-functioning health system and that a minimum of eight ANC visits has been shown to increase women’s satisfaction with ANC [41]. In addition, the WHO emphasize that ANC providers must have good clinical and communication skills. The development of adequate skills to establish a trusting relationship and facilitate an open dialogue with women with traumatic childhood experiences may require post-graduate training for doctors, midwives and nurses, as these skills may not have been cultivated previously. The WHO also recommends midwifery-led continuity-of-care models (caseload midwifery), in which, for example, a small group of two or three midwives working in teams provide antenatal care for a number of women. A study comparing standard care with caseload midwifery for women with complex social factors showed that the women in the caseload group were more likely to be referred to multidisciplinary support services [42]. Furthermore, caseload midwifery for vulnerable women has been shown to increase trust, promote women’s active engagement during ANC visits, including their participation in decision-making processes and enhance the provision of individualised care [43,44,45].

### Strengths and Limitations

The women were recruited from an NGO offering counseling and therapy for adults growing up with alcoholic parents. Furthermore, the women’s educational levels were high and 11 out of 12 women cohabited with a male partner. This is liable to pose a selection bias, as these women may have been more resourceful than women growing up with alcoholic parents in general. Most of the women were expecting their first child, which may have affected their experiences, as well as their concerns. In addition, more women had grown up with an alcoholic father rather than an alcoholic mother; a previous study showed that mental health problems are more pronounced among the daughters of alcoholic mothers compared to the daughters of alcoholic fathers [5]. These factors all affected the diversity of the sample and, thus, the transferability of the study’s findings to the overall population group [25]. Furthermore, a total of 12 participants is a limited number with which to explore a phenomenon. This affects the robustness of the study’s conclusion.

At the same time, there are also several strengths related to the study. One strength is that the main and sub-categories were extensively discussed among all the authors. This increased the reliability of the study findings [46]. Another strength of this study is the length of the interviews, which increased the information power in the overall dataset [47]. Furthermore, we were successful at recruiting women with traumatic childhood experiences, considering the known difficulties involved in recruiting vulnerable populations [25]. Finally, to the authors’ knowledge, very few qualitative studies have previously focused on the pregnant daughters of alcoholics.

## 5. Conclusions

This small-scale study makes a new contribution to the limited knowledge base regarding how adult daughters’ experiences during pregnancy can be impacted by growing up with alcoholic parents.

The upbringing of the women in this study affected their trust in others. A limited number of friends and strained relationships with their parents impacted the women’s access to information and emotional support during their pregnancy negatively. The women mainly relied on their partners, who played a pivotal role in their mental health throughout the pregnancy period, especially in situations perceived to be challenging or concerning by the women.

The ANC visits mainly focused on the physical aspects of pregnancy. During the visits, the women were not asked by their ANC providers about circumstances pertaining to their upbringing, nor did the women provide this information themselves. The women described stress and concerns in relation to the baby’s health, lack of predictability and control during the pregnancy period and apprehensiveness regarding both the birth and their motherhood ability following birth.

The implications of this study for practice include systematic screening for traumatic childhood experiences among all pregnant women upon entrance to ANC. Furthermore, the post-graduate training of ANC providers in how to establish trust and communicate with these women may be needed, as well as sufficient time resources and frequency of visits. Antenatal preparation classes may serve as important sources of informational and social support for this group of women. Finally, as women’s emotional vulnerabilities are likely to continue after birth, parenting courses may aid women in establishing healthy parenting models, despite their adverse childhood experiences.

## Data Availability

According to the General Data Protection Regulation, the qualitative data are confidential and cannot be provided.
